# Dual-Layer Factor-Graph Optimization for Delayed Star-Tracker/IMU Fusion in Highly Dynamic Spacecraft Attitude Estimation

**DOI:** 10.3390/s26134155

**Published:** 2026-07-01

**Authors:** Chao Zhang, Yanjun Yu, Huayi Li

**Affiliations:** School of Astronautics, Harbin Institute of Technology, Harbin 150001, China; 21b918083@stu.hit.edu.cn (C.Z.); lihuayi@hit.edu.cn (H.L.)

**Keywords:** spacecraft attitude estimation, factor graph optimization, delayed measurement, asynchronous sensor fusion

## Abstract

Accurate attitude estimation for highly dynamic spacecraft relies on robust fusion of star-tracker and inertial measurements. However, asynchronous sensing, motion blur in star images, and delayed star-tracker outputs can significantly degrade estimation accuracy and temporal consistency. To address these challenges, this paper proposes a dual-layer factor graph optimization framework for asynchronous star-tracker/IMU fusion under highly dynamic conditions. At the lower layer, high-rate IMU measurements are combined with motion-blurred star streak observations to construct a local factor graph over the exposure interval. The proposed local fusion process reconstructs discrete star-trail points, estimates angular velocity, and selects IMU-aligned representative observations for temporally consistent association of blurred star measurements. At the upper layer, delayed attitude constraints, propagated star-vector information, and inertial rotational constraints are jointly incorporated to refine the attitude trajectory. Simulation and semi-physical experimental results demonstrate that the proposed framework achieves higher estimation accuracy, stronger robustness, and better tolerance to delayed or intermittent star-tracker observations than the comparison methods, while maintaining practical computational efficiency for near-real-time onboard implementation.

## 1. Introduction

To achieve high-precision on-orbit attitude determination, spacecraft commonly rely on multi-sensor fusion to obtain reliable attitude information [1,2]. Among various attitude sensors, inertial measurement units (IMUs) and star trackers are widely adopted due to their low cost, compact size, and broad applicability [3,4]. Their complementary characteristics enable accurate and reliable attitude estimation across diverse mission scenarios, including highly dynamic operating conditions [5,6].

In practical high-dynamic environments, spacecraft are often required to perform rapid maneuvers, aggressive rotations, or high-speed transfers in response to mission demands [7,8]. Under such conditions, the finite exposure time of star trackers causes motion blur during image acquisition, resulting in elongated star streaks rather than clear point features [9]. Furthermore, the subsequent processing pipeline, including image restoration [10], star identification [11], matching, and attitude determination [12,13], introduces non-negligible processing latency.

Meanwhile, although IMUs provide high-rate rotational measurements, their outputs inevitably suffer from bias drift and accumulation errors [14]. Owing to the complementary characteristics of the two sensors, the fusion of star trackers and IMUs has been widely investigated for accurate and robust attitude estimation [15,16,17]. Existing fusion approaches can generally be classified into loosely coupled [18] and tightly coupled methods [19]. Loosely coupled methods combine high-level outputs from individual sensors with relatively simple structures, but their performance may degrade significantly when sensor quality deteriorates or measurements become unreliable [20]. In contrast, tightly coupled methods [21] directly utilize low-level sensor measurements and often integrate online calibration or measurement evaluation into the optimization process, thereby improving robustness under incomplete or degraded observations [22,23]. For example, star vectors extracted from star-tracker observations can be fused with IMU angular rate measurements through preintegration while simultaneously compensating for IMU bias [24,25].

However, in highly dynamic scenarios, motion blur severely degrades star image quality, making accurate extraction of star vectors from a single frame difficult and time-consuming. Consequently, star-tracker outputs are often delayed relative to high-rate IMU measurements, resulting in significant sensing asynchrony [24]. In addition, multiple IMU measurements are generated within a single exposure interval, making it challenging to determine which IMU state should be associated with the processed star observation. Even with hardware synchronization mechanisms, operation may still introduce temporal misalignment due to clock drift and update inconsistencies, further complicating accurate time alignment between the two sensors [26].

Existing approaches for handling delayed measurements and temporal misalignment in multi-sensor fusion systems can generally be categorized into hardware-based synchronization and software-based temporal alignment methods. Nikolić et al. [27] developed a visual–inertial system based on field-programmable gate array (FPGA) triggering to achieve hardware-level synchronization. However, such approaches are often difficult to integrate and may suffer from trigger instability under harsh operating conditions, such as strong radiation or severe vibration. In addition, star trackers in space applications may become intermittently unavailable due to lighting conditions, pointing constraints, or environmental interference. Under these conditions, fixed hardware synchronization mechanisms cannot flexibly adapt to varying exposure settings.

To address temporal misalignment at the software level, Qing et al. [26] proposed an optimization-based online temporal calibration method within a visual–inertial odometry (VIO) framework, where feature velocity was used to estimate a constant temporal offset. However, practical sensing delays may vary over time, limiting the validity of the constant-offset assumption. Song et al. employed cubic spline interpolation for translational motion and spherical linear interpolation (SLERP) for rotational states. Although interpolation improves temporal continuity, the resulting trajectories may not strictly satisfy the true spacecraft dynamic constraints, especially when different sensing modalities evolve at significantly different rates.

Liu et al. [28] incorporated a temporal offset variable into a Multi-State Constraint Kalman Filter (MSCKF) framework by decoupling the Jacobian from the system state. Although this formulation simplifies implementation, it still implicitly depends on temporally aligned reference measurements obtained through prediction or interpolation, which may introduce additional uncertainty. Kahraman et al. [29] addressed delayed star-tracker measurements by propagating the system state to the current time using dynamic models. However, repeated state propagation may alter the original measurement information and lead to accumulated estimation errors. Fundamentally, these approaches alleviate temporal asynchrony through model-based alignment rather than directly incorporating delayed measurements into the estimation framework.

Factor graph optimization (FGO) has emerged as an effective framework for multi-sensor navigation and state estimation because it enables the fusion of heterogeneous measurements within a unified probabilistic optimization framework while maintaining global consistency. For example, Wen et al. [30] combined GNSS and INS measurements within an FGO framework and improved positioning robustness by assigning different weights to line-of-sight (LOS) and non-line-of-sight (NLOS) observations. Shan et al. [31] proposed a tightly coupled lidar–inertial FGO framework that utilized IMU measurements for motion compensation and bias estimation, significantly improving state estimation accuracy. Lyu et al. [32] incorporated inertial, position, velocity, and attitude constraints into a unified factor graph for spacecraft navigation while accounting for Earth-rotation and navigation-frame effects. These studies demonstrate the effectiveness of FGO for robust sensor fusion. Building upon these advances, this paper adopts a factor graph formulation to address asynchronous star-tracker/IMU fusion and delayed measurement processing under motion blur conditions.

This paper proposes a dual-layer factor graph framework for asynchronous star-tracker/IMU fusion under motion blur and delayed observations. Instead of relying on explicit interpolation, retrospective propagation of delayed measurements, or continuous-time offset estimation, the proposed framework combines a lower-layer local fusion graph with an upper-layer global optimization graph. The lower layer extracts motion-related information from blurred star streak observations and high-rate IMU measurements, while the upper layer refines the attitude trajectory using delay-aware star-tracker constraints and inertial factors.

The main contributions of this paper are summarized as follows:

- A lower-layer local factor graph formulation for motion-blurred star images is proposed, in which point-wise motion constraints along star streak trajectories are jointly optimized with IMU measurement constraints to estimate star feature coordinates and angular velocity.
- An upper-layer factor graph formulation with attitude-delay factors is proposed for global attitude refinement under highly dynamic conditions. It incorporates high-disturbance inertial rotational constraints and star-vector factors to account for processing delays.
- A dual-layer coupled fusion strategy is proposed, where the lower layer performs per-frame local optimization and provides optimized feature coordinates and angular velocity estimates, while the upper layer conducts global attitude optimization. Simulation and semi-physical experiments demonstrate the effectiveness and robustness of the proposed approach under varying blur levels, pixel noise, and timing misalignment.

The remaining sections of this work are structured as follows. Section 2 introduces the imaging model for highly dynamic situations and the IMU-based attitude measuring approach. Section 3 describes the fusion of IMU and star-trajectory data, as well as the proposed bottom-level factor graph optimization methodology. Section 4 describes the global attitude estimate approach using factor graph optimization. Section 5 evaluates and reports the results of the numerical simulation and semi-physical experiments. Section 6 summarizes the article and outlines next research topics.

## 2. Problem Description and Model

During high-velocity spacecraft operations, the platform continues to spin throughout the camera exposure period. As a result, each star point suffers noticeable motion on the picture plane instead of appearing as a static point, as in normal low-dynamic situations [33]. Long-term spacecraft operation adds non-negligible IMU bias to gyroscope readings [34].

Both influences must be explicitly included in the modeling and optimization framework to enable proper attitude assessment.

### 2.1. Image Trajectory Degradation Model

Assume that the coordinate of a star point in the camera frame is ${\left[\;x_{c},\;y_{c},\;z_{c}\;\right]}^{T}$. Its projection onto the two-dimensional image plane is denoted as ${\left[x_{i},\;y_{i}\right]}^{T}$. The projection relationship is given by:

(1) $$x_{i}=-\frac{fx_{c}}{z_{c}},\;y_{i}=-\frac{fy_{c}}{z_{c}}.$$

where *f* denotes the focal length of the camera.

During the exposure interval, if the spacecraft undergoes continuous high-rate rotation, the star point moves across the image plane, forming a streak rather than a static point. Let the position of the star in the camera frame at time *t* be ${\left[\;x_{c}^{t},\;y_{c}^{t},\;z_{c}^{t}\;\right]}^{T}$. At time t+Δt, its position becomes ${\left[\;x_{c}^{t+\Delta t},\;y_{c}^{t+\Delta t},\;z_{c}^{t+\Delta t}\;\right]}^{T}$, which satisfies

(2) $$\left[\begin{array}{c} x_{c}^{t+\Delta t}\\ y_{c}^{t+\Delta t}\\ z_{c}^{t+\Delta t} \end{array}\right]=\;R\left[\begin{array}{c} x_{c}^{t}\\ y_{c}^{t}\\ z_{c}^{t} \end{array}\right],$$

where R denotes the relative rotation over the interval Δt. Since the star coordinates are expressed in the rotating camera frame [35], the relative motion of a star is described by

(3) $$R=I-{\left[\omega \right]}_{\times }\;\Delta t,$$

where the skew-symmetric matrix ${\left[\omega \right]}_{\times }$ is defined as

(4) $${\left[\omega \right]}_{\times }=\left[\begin{array}{ccc} 0 & -\omega_{z} & \omega_{y}\\ \omega_{z} & 0 & -\omega_{x}\\ -\omega_{y} & \omega_{x} & 0 \end{array}\right],$$

where $\omega_{x}$, $\omega_{y}$, and $\omega_{z}$ are the three components of the angular velocity vector ω.

Using the skew-symmetric matrix representation of the angular velocity, the time derivative of a point under rotational motion can be written as

(5) $$\begin{array}{cc} \frac{d}{dt}\left[\begin{array}{c} x_{c}\\ y_{c}\\ z_{c} \end{array}\right] & =-{\left[\omega \right]}_{\times }\left[\begin{array}{c} x_{c}\\ y_{c}\\ z_{c} \end{array}\right] \end{array}.$$

Based on the projection Equation (1) and the rotational motion model in Equation (5), the image plane motion of a star point during the exposure interval Δt can be approximated by

(6) $$x_{i}^{t+\Delta t}=x_{i}^{t}+y_{i}^{t}\;\omega_{z}^{t}\;\Delta t+f\;\omega_{y}^{t}\;\Delta t,$$

(7) $$y_{i}^{t+\Delta t}=y_{i}^{t}-x_{i}^{t}\;\omega_{z}^{t}\;\Delta t-f\;\omega_{x}^{t}\;\Delta t.$$

The above formulation characterizes the coordinate variation of a star point within a single-frame streak image under rotational motion.

### 2.2. IMU-Based Attitude Measurement Model

A MEMS gyroscope provides measurements of angular velocity affected by bias and noise:

(8) $${\tilde{\omega }}_{k}=\omega_{k}+b^{g}+\eta^{g},$$

where ω is the true angular velocity, $b^{g}$ denotes the gyroscope bias, and $n^{g}∼N\left(0,\Sigma_{g}\right)$.

The state at time t+Δt can be obtained by integrating $\dot{R}=R{\left[\omega \right]}_{\times }$:

(9) $$R\left(t+\Delta t\right)=R\left(t\right)\;\mathrm{Exp}\;\left(\left({\tilde{\omega }}_{k}-b^{g}-\eta^{g}\right)\Delta t\right).$$

The accumulated relative rotation from frame *i* to frame *j* is given by

(10) $$\Delta R_{ij}≐R_{i}^{⊤}R_{j}=\prod_{k=i}^{j-1}\mathrm{Exp}\;\left(\left({\tilde{\omega }}_{k}-b_{i}^{g}-\eta_{k}^{g}\right)\Delta t\right).$$

Following the Lie-group perturbation framework on SO(3) presented in [36], the rotational noise can be separated from the nominal rotation increment through first-order perturbation analysis. This derivation relies on the conjugation property of the matrix exponential:

(11) $$\mathrm{Exp}\left(\phi \right)\;R=R\;\mathrm{Exp}\;\left(R^{⊤}\phi \right),$$

Applying Equation (11) to the relative rotation model in Equation (10), the rotational noise can be isolated from the nominal rotation increment, yielding

(12) $$\begin{array}{cc} \Delta R_{ij}\; & \approx \prod_{k=i}^{j-1}\mathrm{Exp}\;\left(({\tilde{\omega }}_{k}-b_{i}^{g})\Delta t\right)\cdot \mathrm{Exp}\;\left(-J_{r}\;\left(({\tilde{\omega }}_{k}-b_{i}^{g})\Delta t\right)\eta_{k}^{g}\Delta t\right)\\ \; & \approx \Delta {\tilde{R}}_{ij}\left(b_{i}^{g}\right)\;\mathrm{Exp}\;\left(-\delta \phi_{ij}\right). \end{array}$$

where $J_{r}\left(\cdot \right)$ denotes the right Jacobian on SO(3) as defined in [36].

## 3. Lower-Layer Local Fusion via IMU–Image Alignment

**Figure 1:**
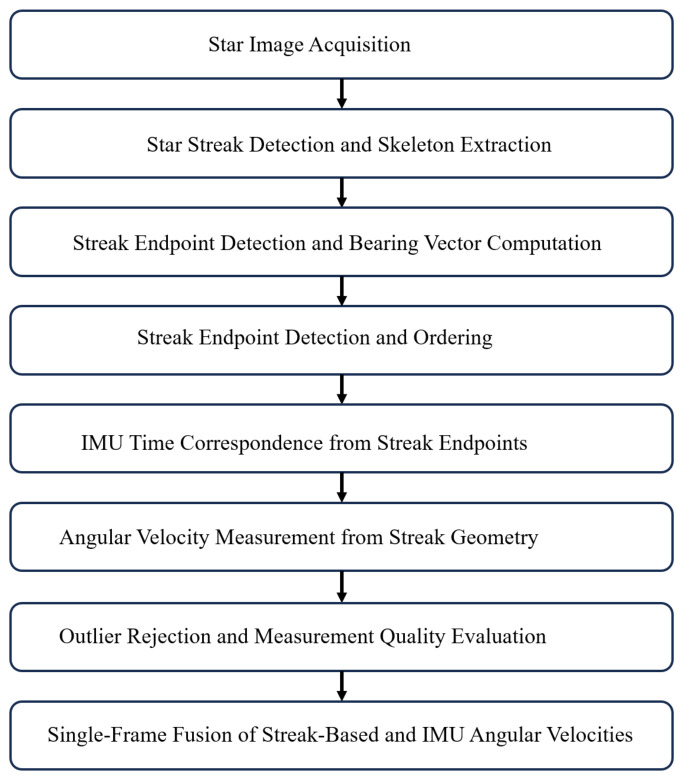
Lower-layer IMU–image alignment and local fusion pipeline over one exposure interval.

During high-speed spacecraft maneuvers, severe motion blur is introduced into star-tracker images, causing point-like stars to appear as elongated streaks on the image plane [37]. Under such conditions, approaches that first restore star streaks into point features inevitably discard valuable motion information encoded along the streak trajectories. To fully exploit this information, the proposed lower-layer framework first extracts the skeleton structure of the blurred star streaks. Based on the star motion model within the exposure interval and the available IMU measurements, corresponding points are generated along the streak trajectories and associated with IMU sampling instants. The overall processing pipeline of the lower layer is illustrated in Figure 1.

**Figure 2:**
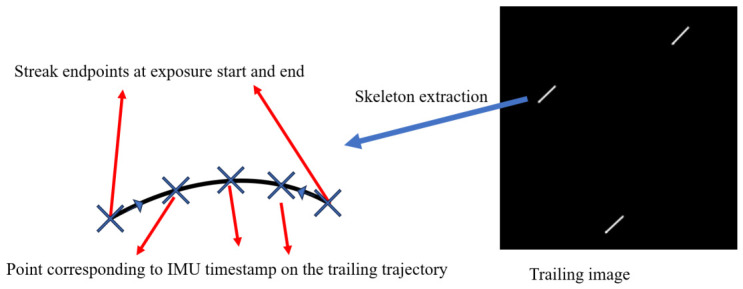
Alignment between skeletonized star streaks and IMU measurements.

Specifically, the two endpoints of each extracted star streak correspond to the known exposure start and end timestamps, as illustrated in Figure 2. By exploiting the star motion model together with the IMU measurements collected during the exposure interval, Equations (6) and (7) are used to propagate these endpoint coordinates to the IMU sampling instants, yielding IMU-aligned points along the streak trajectory. These aligned points subsequently serve as measurements of streak point coordinates and angular velocity in the local optimization.

Since these propagated quantities are derived from raw gyroscope measurements, they inevitably inherit errors introduced by sensor noise and gyroscope biases. Therefore, a local factor graph optimization is subsequently performed to jointly refine the streak point coordinates, angular velocity, and gyroscope bias by incorporating additional constraints.

### 3.1. Streak Kinematic Constraints

Let the image coordinates be $\left\{x_{i},y_{i}\right\}$, and define the corresponding image plane velocities as

(13) $$\begin{array}{cc} v_{x} & ={\dot{x}}_{i},\\ v_{y} & ={\dot{y}}_{i}. \end{array}$$

Combining the above relationship with Equation (1) yields

(14) $$\begin{array}{cc} v_{x} & =-f\;\left(\frac{{\dot{x}}_{c}}{z_{c}}-\frac{x_{c}{\dot{z}}_{c}}{{\dot{z}}_{c}^{2}}\right),\\ v_{y} & =-f\;\left(\frac{{\dot{y}}_{c}}{z_{c}}-\frac{y_{c}{\dot{z}}_{c}}{{\dot{z}}_{c}^{2}}\right). \end{array}$$

Substituting Equation (14) into Equations (4) and (5) gives

(15) $$\begin{array}{c} \left[\begin{array}{c} V_{x}\\ V_{y} \end{array}\right]=f\left[\begin{array}{ccc} -\frac{x_{i}y_{i}}{f^{2}} & \frac{x_{i}^{2}}{f^{2}}+1 & \frac{y_{i}}{f}\\ -\left(\frac{y_{i}^{2}}{f^{2}}+1\right) & \frac{x_{i}y_{i}}{f^{2}} & -\frac{x_{i}}{f} \end{array}\right]\left[\begin{array}{c} \omega_{x}\\ \omega_{y}\\ \omega_{z} \end{array}\right]. \end{array}$$

To estimate the angular velocity from blurred trajectories, the image plane motion is related to the corresponding angular velocity through local streak constraints. Assume that *N* blurred star streaks are detected in one exposure interval. For each streak *n* (n=1,…,N), *m* image samples are associated with the corresponding IMU samples according to temporal order, with Δt denoting the sampling interval between adjacent points.

Based on Equation (15), the image plane velocity observations of the *n*-th streak can be expressed in matrix form as

(16) $$v^{\left(n\right)}\Delta t=H^{\left(n\right)}\omega \;\Delta t,$$

where

(17) $$v^{\left(n\right)}=\left[\begin{array}{c} v_{x,1}^{\left(n\right)}\\ v_{y,1}^{\left(n\right)}\\ \vdots \\ v_{x,m}^{\left(n\right)}\\ v_{y,m}^{\left(n\right)} \end{array}\right],\;\omega =\left[\begin{array}{c} \omega_{x}\\ \omega_{y}\\ \omega_{z} \end{array}\right],$$

and $H^{\left(n\right)}\in R^{2m\times 3}$ is given by

(18) $$H^{\left(n\right)}=\left[\begin{array}{ccc} -\frac{x_{1}^{\left(n\right)}y_{1}^{\left(n\right)}}{f} & \frac{{\left(x_{1}^{\left(n\right)}\right)}^{2}}{f}+f & -y_{1}^{\left(n\right)}\\ -\frac{{\left(y_{1}^{\left(n\right)}\right)}^{2}}{f}-f & \frac{x_{1}^{\left(n\right)}y_{1}^{\left(n\right)}}{f} & x_{1}^{\left(n\right)}\\ \vdots & \vdots & \vdots \\ -\frac{x_{m}^{\left(n\right)}y_{m}^{\left(n\right)}}{f} & \frac{{\left(x_{m}^{\left(n\right)}\right)}^{2}}{f}+f & -y_{m}^{\left(n\right)}\\ -\frac{{\left(y_{m}^{\left(n\right)}\right)}^{2}}{f}-f & \frac{x_{m}^{\left(n\right)}y_{m}^{\left(n\right)}}{f} & x_{m}^{\left(n\right)} \end{array}\right].$$

Similarly, using Equations (6) and (7), the displacement observations of the same streak can be written as

(19) $$\Delta u^{\left(n\right)}=G^{\left(n\right)}\omega ,$$

where

(20) $$\Delta u^{\left(n\right)}=\left[\begin{array}{c} \Delta x_{1}^{\left(n\right)}\\ \Delta y_{1}^{\left(n\right)}\\ \vdots \\ \Delta x_{m-1}^{\left(n\right)}\\ \Delta y_{m-1}^{\left(n\right)} \end{array}\right],$$

with

(21) $$\Delta x_{k}^{\left(n\right)}=x_{k+1}^{\left(n\right)}-x_{k}^{\left(n\right)},\;\Delta y_{k}^{\left(n\right)}=y_{k+1}^{\left(n\right)}-y_{k}^{\left(n\right)},$$

and

(22) $$G^{\left(n\right)}=\left[\begin{array}{ccc} 0 & f\Delta t & y_{1}^{\left(n\right)}\Delta t\\ -f\Delta t & 0 & -x_{1}^{\left(n\right)}\Delta t\\ \vdots & \vdots & \vdots \\ 0 & f\Delta t & y_{m-1}^{\left(n\right)}\Delta t\\ -f\Delta t & 0 & -x_{m-1}^{\left(n\right)}\Delta t \end{array}\right]\in R^{2(m-1)\times 3}.$$

The velocity-based and displacement-based observations provide complementary constraints on the angular velocity. Therefore, they are combined into a unified observation model

(23) $$z^{\left(n\right)}=M^{\left(n\right)}\omega ,$$

where

(24) $$z^{\left(n\right)}=\left[\begin{array}{c} v^{\left(n\right)}\Delta t\\ \Delta u^{\left(n\right)} \end{array}\right],\;M^{\left(n\right)}=\left[\begin{array}{c} H^{\left(n\right)}\Delta t\\ G^{\left(n\right)} \end{array}\right].$$

The unified observation model in Equation (23) is employed as the motion constraint in the lower-layer factor graph optimization.

### 3.2. Lower-Layer Factor Graph Formulation

**Figure 3:**
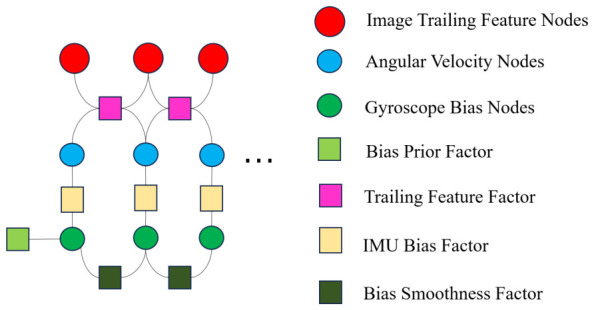
Factor graph structure of the lower-layer local optimization over one exposure interval.

A local factor graph is constructed over one exposure interval. The lower-layer state is defined as

(25) $$X_{\mathrm{low}}=\left\{\left\{u_{k}^{\left(n\right)}\right\},\left\{\omega_{k}\right\},\left\{b_{k}^{g}\right\}\right\},$$

where $u_{k}^{\left(n\right)}$ denotes the sampled image-point positions along the *n*-th streak, $\omega_{k}$ is the angular velocity at IMU sampling instant *k*, and $b_{k}^{g}$ is the gyroscope bias within the same exposure interval. Figure 3 shows the corresponding factor graph structure.

The consistency between the angular velocity and gyroscope measurement is modeled by

(26) $$r_{\omega ,k}=\omega_{k}^{g}-\omega_{k}-b_{k}^{g},$$

where $\omega_{k}^{g}$ is the gyroscope measurement at time index *k*.

Within one exposure interval, the gyroscope bias is assumed to vary slowly and is modeled as a random walk:

(27) $$r_{b,k}=b_{k+1}^{g}-b_{k}^{g},\;r_{b,k}∼N\left(0,\Sigma_{b}\Delta t\right),$$

where $\Sigma_{b}$ is the covariance of the gyroscope bias random walk.

The lower-layer objective is formulated as the weighted sum of the star streak geometric residuals, the angular velocity consistency residuals, and the bias evolution residuals:

(28) $$\min_{X_{\mathrm{low}}}\;\sum_{n\in N}{∥r^{\left(n\right)}∥}_{\Sigma_{n}^{-1}}^{2}+\sum_{k\in K}{∥r_{\omega ,k}∥}_{\Sigma_{\omega }^{-1}}^{2}+\sum_{k\in K}{∥r_{b,k}∥}_{\Sigma_{b}^{-1}}^{2},$$

where N denotes the set of detected streaks in the current exposure interval, and K denotes the set of IMU sampling indices within that interval.

By solving Equation (28), the lower layer produces locally refined angular motion estimates together with representative streak observations for subsequent upper-layer time alignment and factor construction.

## 4. Global Attitude Optimization Layer with Delay-Compensated Star-Tracker Factors

This section presents the upper-layer global attitude optimization framework in the proposed dual-layer architecture. Building on the lower-layer local fusion results, the upper layer constructs a factor graph optimization framework that jointly incorporates inertial rotation constraints, representative star-observation constraints, and delayed star-tracker attitude measurements. The resulting formulation enables temporally consistent attitude refinement under asynchronous sensing conditions without introducing an additional continuous-time trajectory model.

### 4.1. Global Attitude Graph Optimization

Following the lower-layer initialization and local fusion of blurred star streak observations with high-rate IMU data, the system proceeds to global attitude optimization over sequential attitude states. In high-dynamic scenarios, rapid spacecraft maneuvers may induce non-negligible gyroscope bias variations, which degrade conventional inertial propagation if not explicitly modeled. To improve long-term consistency and suppress inertial drift, the upper layer jointly optimizes the retained attitude states and gyroscope bias states by minimizing all factor residuals over the retained optimization states.

The upper-layer optimization problem is formulated as

(29) $$\begin{array}{cc} \min_{X}\;\; & \sum_{k=0}^{N-1}{∥r_{\Delta R_{ij}}∥}_{\Sigma_{r_{ij}}}^{2}\\ \; & \;+\sum_{l\in R(C)}∥r_{l}^{★}{∥}_{\Sigma_{C}}^{2}+\sum_{m\in S}{∥r_{\phi_{m}}∥}_{\Sigma_{S}}^{2}, \end{array}$$

where $r_{\Delta R_{ij}}$ is the rotation integration residual derived from IMU measurements, $r_{l}^{★}$ is the star-vector residual constructed from the representative star observations provided by the lower layer, and $r_{\phi_{m}}$ is the delayed attitude residual associated with the star-tracker attitude output. The matrices $\Sigma_{r_{ij}}$, $\Sigma_{C}$, and $\Sigma_{S}$ denote the corresponding information matrices.

The state vector X contains the retained attitude nodes and the associated gyroscope bias states. The upper layer is activated after a valid star-based attitude prior becomes available, and is subsequently updated whenever sufficient delayed star-related constraints have been accumulated.

#### 4.1.1. Rotation Integration Constraint Under Large IMU Bias Disturbances

Within each lower-layer factor graph optimization associated with a single image exposure interval, the IMU bias is assumed to be locally constant due to the short exposure duration. Since each lower-layer optimization is performed independently for a single image interval, no long-term bias evolution is modeled. Long-term bias variations are explicitly taken into account in the upper-layer global optimization. To account for bias-dependent inertial propagation, the gyroscope bias is linearized around a nominal value ${\bar{b}}_{i}^{g}$, and the current bias estimate is represented as

(30) $$b_{i}^{g}={\bar{b}}_{i}^{g}+\delta b_{i}^{g},$$

where $\delta b_{i}^{g}$ denotes the bias correction with respect to the linearization point.

Accordingly, the bias-corrected preintegrated rotation measurement can be written as

(31) $$\begin{array}{cc} \Delta {\tilde{R}}_{ij}\left(b_{i}^{g}\right)\; & =\prod_{k=i}^{j-1}\mathrm{Exp}\;\left(\left({\tilde{\omega }}_{k}-b_{i}^{g}\right)\Delta t\right)\\ \; & =\prod_{k=i}^{j-1}\mathrm{Exp}\;\left(\left({\tilde{\omega }}_{k}-{\bar{b}}_{i}^{g}-\delta b_{i}^{g}\right)\Delta t\right). \end{array}$$

Define the nominal preintegrated rotation at the linearization point ${\bar{b}}_{i}^{g}$ as

(32) $$\Delta {\bar{R}}_{ij}\;≐\;\prod_{k=i}^{j-1}\mathrm{Exp}\;\left(\left({\tilde{\omega }}_{k}-{\bar{b}}_{i}^{g}\right)\Delta t\right).$$

Using the standard first-order bias correction in IMU preintegration, the preintegrated rotation is approximated as

(33) $$\Delta {\tilde{R}}_{ij}\left(b_{i}^{g}\right)\approx \Delta {\bar{R}}_{ij}\;\mathrm{Exp}\;\left(J_{\Delta R_{ij}}^{b_{g}}\;\delta b_{i}^{g}\right),$$

where $J_{\Delta R_{ij}}^{b_{g}}\in R^{3\times 3}$ is the Jacobian of the preintegrated rotation with respect to the gyroscope bias, evaluated at the linearization point. It is given by

(34) $$\begin{array}{cc} J_{\Delta R_{ij}}^{b_{g}}\; & \;≐\;-\sum_{k=i}^{j-1}{\left(\Delta {\bar{R}}_{k+1,j}\left({\bar{b}}_{i}^{g}\right)\right)}^{⊤}J_{r}^{k}\;\Delta t,\\ J_{r}^{k}\; & \;≐\;J_{r}\;\left(\left({\tilde{\omega }}_{k}-{\bar{b}}_{i}^{g}\right)\Delta t\right). \end{array}$$

The rotational residual is defined on SO(3) as

(35) $$r_{\Delta R_{ij}}\;≐\;\mathrm{Log}\;\left(\Delta {\tilde{R}}_{ij}{\left(b_{i}^{g}\right)}^{⊤}R_{i}^{⊤}R_{j}\right).$$

For residual linearization and subsequent Jacobian derivation, the attitude states are parameterized using left-multiplicative perturbations:

(36) $$R_{i}\leftarrow R_{i}\mathrm{Exp}\left(\delta \phi_{i}\right),\;R_{j}\leftarrow R_{j}\mathrm{Exp}\left(\delta \phi_{j}\right),$$

where $\delta \phi_{i},\delta \phi_{j}\in R^{3}$ are local perturbations on SO(3). The gyroscope bias correction is updated additively as

(37) $$\delta b_{i}^{g}\leftarrow \delta b_{i}^{g}+\tilde{\delta }b_{i}^{g},$$

where $\tilde{\delta }b_{i}^{g}\in R^{3}$ is the current optimization increment.

Substituting the perturbation of $R_{i}$ into Equation (35) yields

(38) $$\begin{array}{cc} r_{\Delta R_{ij}}\left(R_{i}\mathrm{Exp}\left(\delta \phi_{i}\right)\right)\; & =\mathrm{Log}\;\left(\Delta {\tilde{R}}_{ij}{\left(b_{i}^{g}\right)}^{⊤}{\left(R_{i}\mathrm{Exp}\left(\delta \phi_{i}\right)\right)}^{⊤}R_{j}\right)\\ \; & =\mathrm{Log}\;\left(\Delta {\tilde{R}}_{ij}{\left(b_{i}^{g}\right)}^{⊤}\mathrm{Exp}\left(-\delta \phi_{i}\right)R_{i}^{⊤}R_{j}\right)\\ \; & \approx r_{\Delta R_{ij}}-J_{r}^{-1}\;\left(r_{\Delta R_{ij}}\right)R_{j}^{⊤}R_{i}\;\delta \phi_{i}. \end{array}$$

Therefore

(39) $$\frac{\partial r_{\Delta R_{ij}}}{\partial \delta \phi_{i}}=-J_{r}^{-1}\;\left(r_{\Delta R_{ij}}\right)R_{j}^{⊤}R_{i}.$$

Similarly, perturbing $R_{j}$ gives

(40) $$\begin{array}{cc} r_{\Delta R_{ij}}\left(R_{j}\mathrm{Exp}\left(\delta \phi_{j}\right)\right)\; & =\mathrm{Log}\;\left(\Delta {\tilde{R}}_{ij}{\left(b_{i}^{g}\right)}^{⊤}R_{i}^{⊤}\left(R_{j}\mathrm{Exp}\left(\delta \phi_{j}\right)\right)\right)\\ \; & \approx r_{\Delta R_{ij}}+J_{r}^{-1}\;\left(r_{\Delta R_{ij}}\right)\delta \phi_{j}. \end{array}$$

Hence

(41) $$\frac{\partial r_{\Delta R_{ij}}}{\partial \delta \phi_{j}}=J_{r}^{-1}\;\left(r_{\Delta R_{ij}}\right).$$

Next, consider the additive perturbation of the gyroscope bias correction

(42) $$\delta b_{i}^{g}\leftarrow \delta b_{i}^{g}+\tilde{\delta }b_{i}^{g}.$$

Using the first-order bias correction model, the rotational residual becomes

(43) $$\begin{array}{cc} r_{\Delta R_{ij}}\left(\delta b_{i}^{g}+\tilde{\delta }b_{i}^{g}\right)\; & =\mathrm{Log}\;\left(\Delta {\tilde{R}}_{ij}\;{\left({\bar{b}}_{i}^{g}+\delta b_{i}^{g}+\tilde{\delta }b_{i}^{g}\right)}^{⊤}R_{i}^{⊤}R_{j}\right)\\ \; & \approx \mathrm{Log}\;(\mathrm{Exp}\;\left(-J_{\Delta R_{ij}}^{b_{g}}\left(\delta b_{i}^{g}+\tilde{\delta }b_{i}^{g}\right)\right)\\ \; & \;\;\Delta {\bar{R}}_{ij}^{⊤}R_{i}^{⊤}R_{j}). \end{array}$$

Applying the first-order approximation gives

(44) $$r_{\Delta R_{ij}}\left(\delta b_{i}^{g}+\tilde{\delta }b_{i}^{g}\right)\approx r_{\Delta R_{ij}}\left(\delta b_{i}^{g}\right)-J_{r}^{-1}\;\left(r_{\Delta R_{ij}}\left(\delta b_{i}^{g}\right)\right)J_{\Delta R_{ij}}^{b_{g}}\;\tilde{\delta }b_{i}^{g}.$$

Therefore, the Jacobian of the rotational residual with respect to the gyroscope bias correction is

(45) $$\frac{\partial r_{\Delta R_{ij}}}{\partial \delta b_{i}^{g}}=-J_{r}^{-1}\;\left(r_{\Delta R_{ij}}\right)J_{\Delta R_{ij}}^{b_{g}}.$$

To model slow bias evolution over the window, the gyroscope bias is further constrained by a random walk prior between adjacent nodes:

(46) $$r_{\Delta b_{ij}^{g}}=b_{j}^{g}-b_{i}^{g}.$$

#### 4.1.2. Inter-Layer Coupling and Discrete Time Alignment

In the proposed dual-layer framework, the lower layer does not propagate a continuous-time trajectory to the upper layer. Instead, each blurred star streak is summarized as a discrete representative observation together with an IMU-aligned timestamp for upper-layer factor construction. Since a star streak is an exposure-integrated measurement, a representative observation must first be selected before introducing star-related constraints into the global graph [38].

For each streak *l*, the effective observation time is selected from the discrete candidate set $T_{l}=\left\{t_{i}\right\}$, which contains the IMU sampling instants within the corresponding exposure interval. The aligned timestamp is determined by

(47) $$t_{l}^{*}=\arg\;\min_{t_{i}\in T_{l}}{∥r_{\omega ,l}\left(t_{i}\right)∥}^{2},$$

where $r_{\omega ,l}\left(t_{i}\right)$ denotes the lower-layer angular motion residual evaluated at candidate time $t_{i}$. Therefore, the proposed alignment is a discrete timestamp assignment on the IMU sampling grid rather than a continuous-time estimation, and no additional temporal state is introduced in the upper layer.

After $t_{l}^{*}$ is selected, the corresponding point on the streak is taken as the representative observation and assigned the timestamp $t_{l}^{*}$. The lower layer then propagates the compact summary

(48) $$Z_{l}=\left\{{\hat{u}}_{l}^{*},\;t_{l}^{*},\;\Lambda_{u,l}^{*}\right\},$$

where ${\hat{u}}_{l}^{*}$ is the representative pixel observation and $\Lambda_{u,l}^{*}$ is its associated information matrix. In the proposed implementation, $t_{l}^{*}$ is treated as a deterministic label inherited from the IMU sampling grid; accordingly, only the observation uncertainty is propagated across layers.

The representative observation selected in the lower layer is transferred to the upper layer together with its associated measurement uncertainty. In the proposed implementation, the lower layer provides the representative pixel observation ${\hat{u}}_{l}^{*}$, the aligned timestamp $t_{l}^{*}$, and the corresponding information matrix $\Lambda_{u,l}^{*}$, which is obtained from the local optimization result.

If the upper-layer star factor is formulated in terms of normalized star-bearing vectors, the representative pixel observation is first mapped to a unit-bearing vector through the camera model, and its uncertainty is propagated by first-order linearization:

(49) $$\Sigma_{b,l}^{*}=J_{b\leftarrow u,l}\;\Sigma_{u,l}^{*}\;J_{b\leftarrow u,l}^{⊤},\;\Lambda_{b,l}^{*}={\left(\Sigma_{b,l}^{*}\right)}^{-1},$$

where $\Sigma_{u,l}^{*}={\left(\Lambda_{u,l}^{*}\right)}^{-1}$, and $J_{b\leftarrow u,l}$ denotes the Jacobian of the pixel-to-bearing mapping evaluated at ${\hat{u}}_{l}^{*}$. In implementation, the resulting uncertainty is represented in the local two-dimensional tangent space of the unit-bearing direction to avoid over-parameterization.

For computational efficiency, only the marginal uncertainty of each representative observation is propagated to the upper layer, while cross-window correlations between different lower-layer summaries are neglected.

#### 4.1.3. Star Vector Factor

Using the inter-layer summary $Z_{l}$ in Equation (48), the upper layer constructs a star-vector factor at the aligned timestamp $t_{l}^{*}$. Let ${\tilde{w}}_{l,t_{l}^{*}}$ denote the measured unit star-bearing vector associated with star *l*. The predicted bearing is given by $R_{t_{l}^{*}}w_{l}$, where $R_{t_{l}^{*}}\in SO\left(3\right)$ is the attitude at the aligned timestamp and $w_{l}\in R^{3}$ is the known inertial-frame direction of star *l*.

To avoid over-parameterization of the unit-norm constraint, the residual is defined on the two-dimensional tangent space of the measured bearing:

(50) $$r_{l}^{★}=P_{l}^{⊤}\left(R_{t_{l}^{*}}w_{l}-{\tilde{w}}_{l,t_{l}^{*}}\right),$$

where $P_{l}\in R^{3\times 2}$ spans the tangent space orthogonal to ${\tilde{w}}_{l,t_{l}^{*}}$, satisfying $P_{l}^{⊤}{\tilde{w}}_{l,t_{l}^{*}}=0$ and $P_{l}^{⊤}P_{l}=I_{2}$. The corresponding factor is weighted by the propagated representative observation uncertainty from the lower layer, i.e., by the tangent space information matrix derived from $\Lambda_{b,l}^{*}$ (or equivalently from $\Lambda_{u,l}^{*}$ in the pixel-domain formulation).

Accordingly, the propagated bearing uncertainty is also represented in the same tangent space. Let $\Sigma_{b,l}^{*}$ denote the bearing-domain covariance obtained from the lower-layer representative observation. Its tangent space covariance and information matrix are given by

(51) $$\Sigma_{\tan,l}^{*}=P_{l}^{⊤}\Sigma_{b,l}^{*}P_{l},\;\Lambda_{\tan,l}^{*}={\left(\Sigma_{\tan,l}^{*}\right)}^{-1}.$$

The resulting factor contribution is therefore weighted as

(52) $$J_{l}^{★}={∥r_{l}^{★}∥}_{\Lambda_{\tan,l}^{*}}^{2}.$$

Unlike continuous-time interpolation or temporal offset estimation methods, the proposed framework directly attaches each representative star observation to the upper-layer graph at the discrete IMU-aligned timestamp selected in the lower layer.

#### 4.1.4. Delayed Attitude Measurement Factor

The delayed attitude factor is constructed from the star-tracker attitude solution, which becomes available after a non-negligible processing delay due to star identification and onboard attitude determination. To ensure temporal consistency under asynchronous sensing, the delayed attitude measurement is inserted at an effective timestamp determined using the same discrete alignment strategy in Equation (47).

Specifically, the delayed attitude measurement is associated with the aligned timestamp $t^{*}$, selected from the IMU sampling grid within the corresponding support interval. The selected $t^{*}$ serves only as the factor insertion time and is not treated as an additional optimization variable.

The residual of the delayed attitude factor is defined as

(53) $$r_{\phi_{m}}=\mathrm{Log}\;\left(R{\left(t^{*}\right)}^{⊤}{\tilde{R}}^{d}\right),$$

where $R(t^{*})$ denotes the estimated attitude at the aligned timestamp $t^{*}$, and ${\tilde{R}}^{d}$ is the delayed attitude measurement provided by the star-tracker.

Compared with the star-vector factor, which is constructed from lower-layer representative observations, the delayed attitude factor directly exploits the complete star-tracker attitude solution after onboard processing. The two factors are therefore complementary in both timing and information granularity.

**Figure 4:**
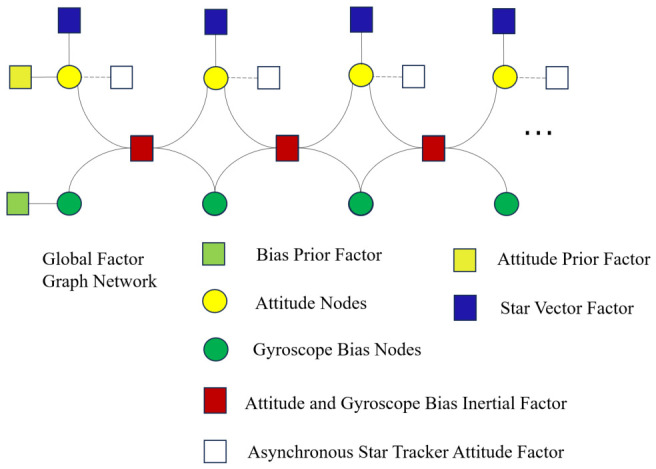
Global attitude factor graph with asynchronous star-tracker constraints.

The overall upper-layer factor graph structure and delayed factor connections are illustrated in Figure 4, where dashed edges indicate delayed measurement constraints.

### 4.2. Dual-Layer Coupled Fusion Strategy

**Figure 5:**
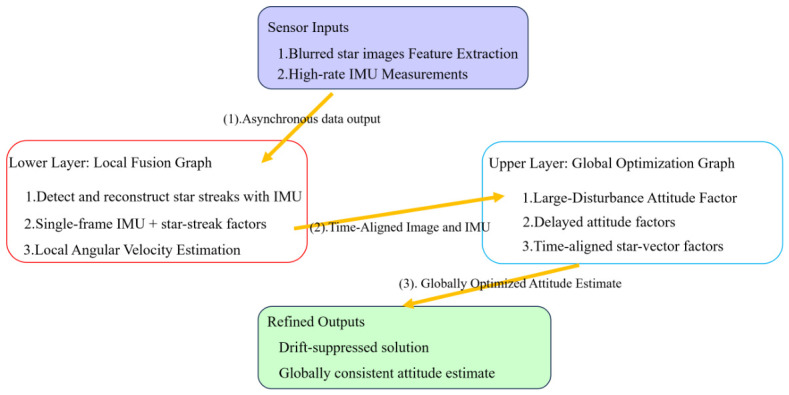
Overview of the proposed dual-layer coupled fusion framework.

To achieve accurate short-term fusion and globally consistent attitude estimation, a dual-layer coupled strategy is adopted, as illustrated in Figure 5, consisting of a lower-layer local factor graph and an upper-layer global factor graph.

The lower layer operates within each image exposure interval and performs local fusion of motion-blurred star streak observations and high-rate IMU measurements. By jointly exploiting star streak motion constraints and IMU observations, the lower-layer graph estimates representative observations associated with IMU-aligned timestamps. The optimization variables mainly include the local angular velocity and star point coordinates.

The upper layer performs global attitude estimation over multiple observation intervals. The graph consists of inertial factors, time-aligned star-vector factors, and delayed attitude factors generated from star identification. The optimization variables include the spacecraft attitude states and gyroscope biases. By jointly optimizing these constraints, the upper layer refines the attitude trajectory and estimates gyroscope biases.

The representative observations and IMU-aligned timestamps estimated by the lower layer are subsequently used to construct the corresponding factors in the upper-layer graph. Once a valid star-based attitude prior becomes available, the upper layer is initialized and performs optimization.

The two layers operate asynchronously. The lower layer is updated whenever valid star streak observations are available, whereas the upper layer is triggered after sufficient observations have been accumulated. In this manner, the lower layer continuously provides observation support for the upper layer, while the upper layer performs global refinement using the accumulated constraints. The factor graph optimization in both layers is solved using the Levenberg–Marquardt algorithm.

## 5. Experiments and Results

The proposed framework is evaluated through numerical simulations and semi-physical experiments. The simulation studies assess estimation accuracy, robustness, and sensitivity under controlled conditions, while the semi-physical experiments validate implementation feasibility, timing behavior, and robustness under realistic sensing and processing constraints.

### 5.1. Simulation Experiments

The simulation studies are conducted based on the Tycho-2 star catalog, with the common parameter settings summarized in Table 1. Unless otherwise specified, the proposed method and the SPKF comparison method [39] use identical sensor parameters, noise covariance settings, sampling configurations, and initial conditions to ensure a fair comparison.

The SPKF is adopted as a representative recursive nonlinear fusion method, where IMU measurements are used for state propagation and star-sensor observations are incorporated at nominal image timestamps for correction. Unlike the proposed factor graph framework, the SPKF does not perform delayed-factor insertion or retrospective smoothing under asynchronous measurements.

**Figure 6:**
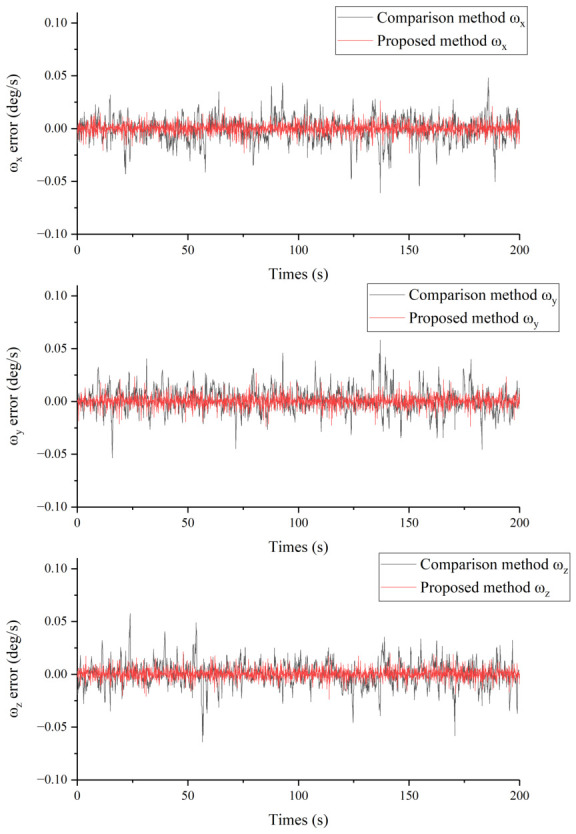
Local angular velocity fusion error for short-tail trajectory (2–2–1 deg/s).

**Figure 7:**
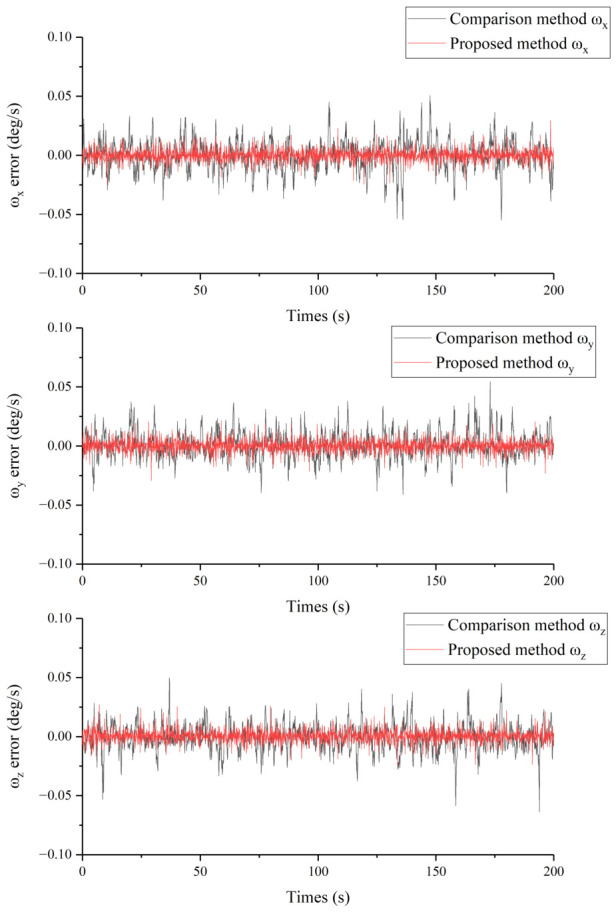
Local angular velocity fusion error for long-tail trajectory (4–4–5 deg/s).

Figure 6 and Figure 7 compare the local angular velocity estimation performance under two motion conditions corresponding to short and long star streaks, i.e., 2–2–1 deg/s and 4–4–5 deg/s, respectively. These two cases are selected because the streak length increases with the angular rate according to Equations (6) and (7).

In both cases, the proposed local factor graph fusion consistently outperforms SPKF on all three axes. For the short-tail case, the proposed method achieves MAE/RMSE values of approximately 0.004–0.006 deg/s, compared with about 0.009–0.012 deg/s for SPKF. For the long-tail case, the proposed method maintains nearly the same error level, whereas the SPKF method remains noticeably worse. These results indicate that the lower-layer formulation is robust to moderate changes in streak length and preserves stable cross-axis consistency through coupled multi-factor fusion.

**Figure 8:**
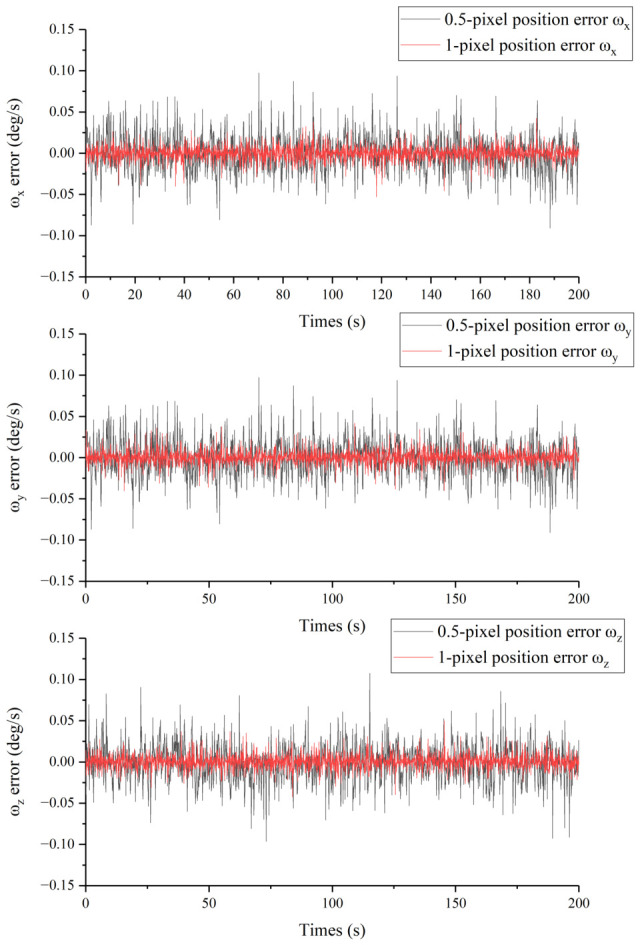
Impact of pixel measurement noise on local angular velocity fusion error under local optimization.

Figure 8 evaluates the sensitivity of the lower-layer optimization to pixel-level star point extraction noise by comparing two noise levels, i.e., 0.5 and 1 pixel. As expected, the angular velocity error increases with pixel noise. When the noise level increases from 0.5 to 1 pixel, the average MAE increases from approximately 0.0066deg/s to 0.0162deg/s, and the average RMSE increases from about 0.0093deg/s to 0.0214deg/s. Although the degradation is evident, the local estimator remains stable under both conditions, indicating good robustness to moderate front-end extraction uncertainty.

**Figure 9:**
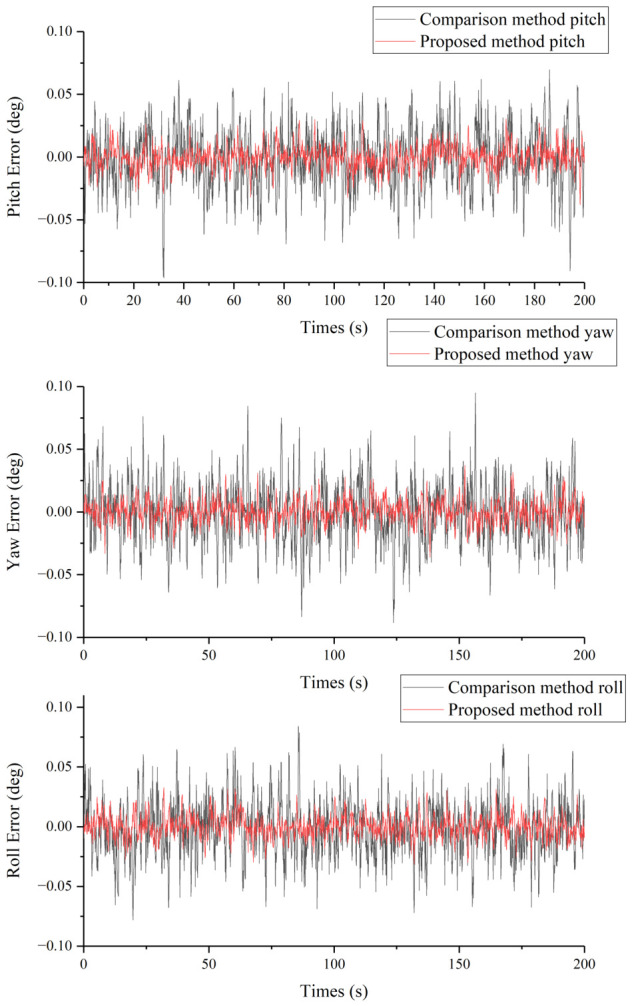
Global attitude estimation errors for the proposed and SPKF.

Figure 9 compares the global attitude estimation performance of the proposed framework and the SPKF. The proposed method achieves an MAE of [0.0068,0.0069,0.0069]deg and an RMSE of [0.0092,0.0093,0.0093]deg, whereas the SPKF yields an MAE of [0.0172,0.0168,0.0167]deg and an RMSE of [0.0230,0.0229,0.0227]deg, respectively. The error reduction is consistent across all three axes, demonstrating that the upper-layer delayed-factor optimization effectively improves global consistency and suppresses long-horizon drift relative to the SPKF. The attitude estimation errors in all three axes remain bounded around zero throughout the entire 200 s simulation period, indicating the absence of observable error accumulation or long-term drift.

**Figure 10:**
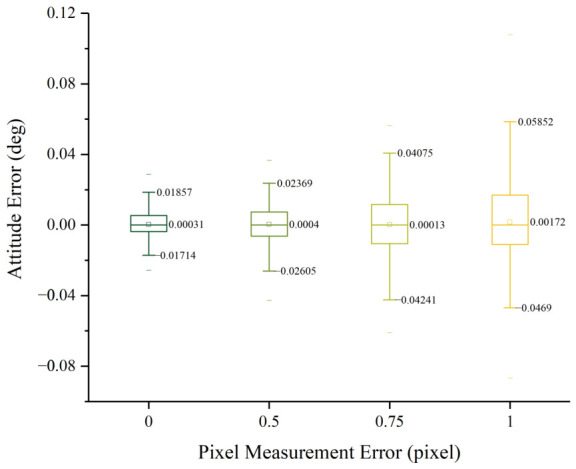
Effect of pixel measurement noise on global attitude estimation errors under global optimization.

Figure 10 shows the global attitude estimation errors under four pixel-level measurement noise conditions (0, 0.5, 0.75, and 1 pixel). As the pixel noise increases, both the median and dispersion of the attitude error increase gradually. The corresponding average MAE/RMSE values increase from 0.0066/0.0094deg at 0 pixel to 0.0188/0.0275deg at 1 pixel. Meanwhile, the mean error remains close to zero in all cases, indicating that the estimator remains approximately unbiased. Overall, the proposed global optimization degrades gracefully as front-end measurement uncertainty increases.

To evaluate robustness against temporal misalignment, timestamp errors of 10, 20, 50, and 100 ms are injected into the delayed attitude and star-vector factors in the upper-layer graph.

**Figure 11:**
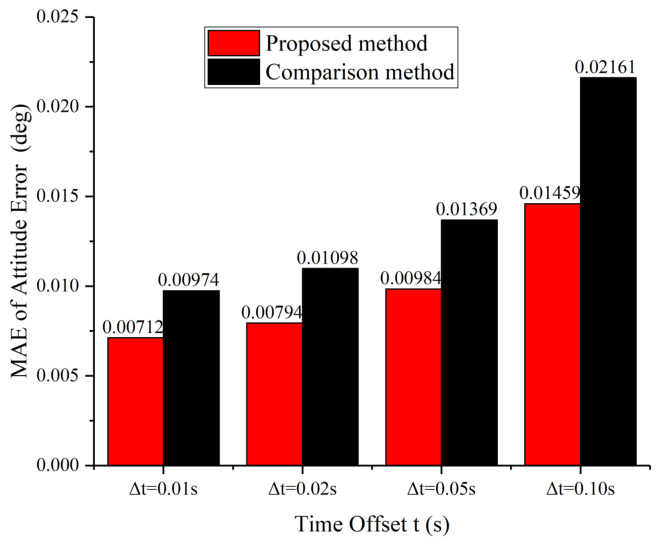
Sensitivity of attitude estimation accuracy to imposed temporal offsets between IMU and star-tracker measurements.

As shown in Figure 11, the estimation error of both methods increases monotonically as the imposed temporal offset between the IMU and image measurements becomes larger. To evaluate the sensitivity of attitude estimation to temporal misalignment, fixed time offsets ranging from 10 ms to 100 ms are intentionally introduced between the star-tracker observations and IMU measurements during fusion. However, the proposed method consistently outperforms SPKF under all tested offset conditions. Its MAE increases from 0.0071deg at a 10 ms offset to 0.0146deg at 100 ms, whereas SPKF increases from 0.0097deg to 0.0216deg. A similar trend is observed for the RMSE. Moreover, the performance gap becomes more pronounced as the temporal offset increases, demonstrating that the proposed framework provides improved robustness to temporal misalignment and asynchronous sensing conditions.

**Figure 12:**
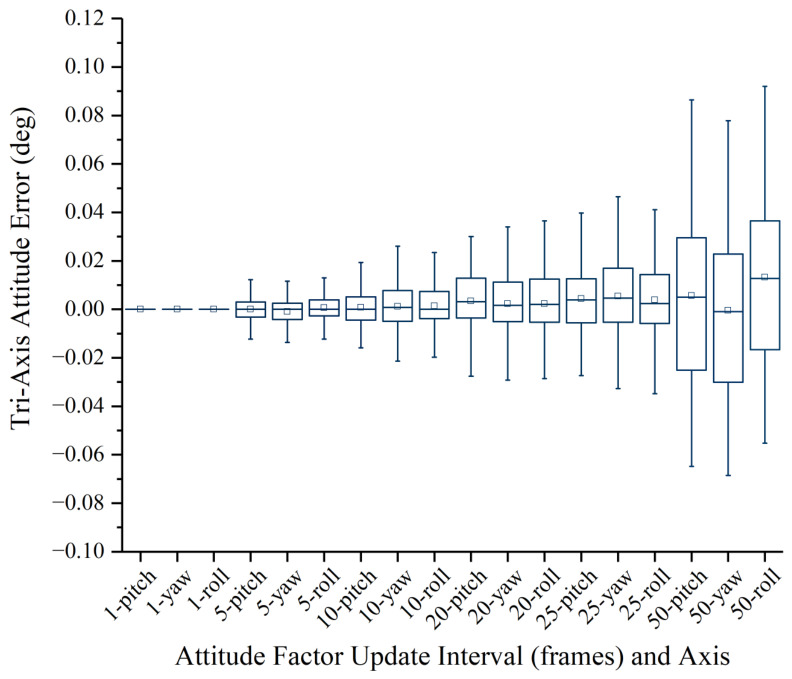
Effect of attitude factor update frequency on global attitude estimation errors under the proposed optimization framework.

Figure 12 evaluates the proposed method under six attitude factor update intervals (1, 5, 10, 20, 25, and 50 frames), which emulate delayed or intermittent availability of star-tracker attitude updates. As the update interval increases, the attitude estimation error grows gradually, indicating weaker absolute correction in the global graph. Nevertheless, the degradation remains smooth and stable, without abrupt divergence. For example, the average MAE/RMSE increases from 0.0044deg/0.0061deg at a 5-frame interval to 0.0302deg/0.0365deg at a 50-frame interval. These results show that the framework remains robust under sparse attitude updates, with the lower layer effectively bridging the intervals between global corrections.

**Figure 13:**
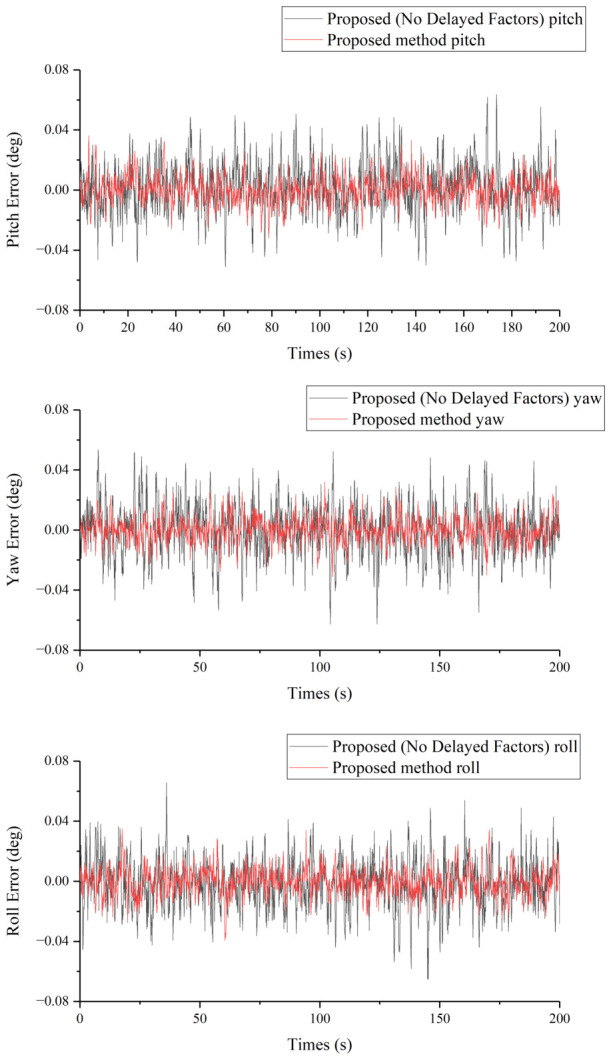
Effect of delayed attitude factors on global attitude estimation performance.

Figure 13 presents an ablation experiment comparing the proposed framework with and without delayed attitude factors during a 200 s simulation. The variant denoted as Proposed (No Delayed Factors) removes the delayed attitude constraints from the upper-layer optimization while retaining all other components unchanged. The results show that incorporating delayed attitude factors significantly improves global attitude estimation accuracy. The proposed method achieves mean three-axis MAE values of approximately 0.0067, 0.0063, and 0.0067deg, with corresponding RMSE values of 0.0094, 0.0085, and 0.0090deg. In contrast, the Proposed (No Delayed Factors)variant exhibits noticeably larger errors, with MAE values increasing to approximately 0.0118, 0.0120, and 0.0117deg, and RMSE values increasing to 0.0158, 0.0164, and 0.0152deg. These results indicate that delayed attitude factors provide effective global temporal constraints for the upper-layer optimization, thereby reducing accumulated attitude drift under asynchronous sensing conditions.

### 5.2. Semi-Physical Experiments

Semi-physical experiments are conducted to validate the proposed framework under realistic sensing and processing conditions. A semi-physical laboratory platform is constructed using a real IMU data, a camera-based image acquisition system for star streak extraction, and an onboard computing unit. The evaluation focuses on angular velocity accuracy, attitude accuracy, gyroscope bias estimation, and computational efficiency.

The platform is intended to emulate the sensing, image-processing, and delayed-observation pipeline under laboratory conditions, rather than a fully coupled spacecraft rotational testbed. Accordingly, the objective of this section is to assess implementation feasibility, timing behavior, and robustness under realistic sensor and processing constraints. The reference attitude and angular rate trajectories used for error evaluation are defined by the predesigned excitation trajectory that synchronously drives the simulated star field playback and the IMU data stream, and are treated as the ground truth in the semi-physical experiments. To further evaluate robustness under temporary degradation of absolute attitude information, additional tests are performed by intentionally interrupting the star-tracker attitude output over specified intervals.

The reference attitude and angular rate trajectories are generated from the known star field playback sequence and the synchronized excitation profile used to drive the IMU stream. Since the platform is intended to emulate the sensing and processing pipeline rather than a fully instrumented rotational truth platform, these references should be interpreted as synchronized nominal trajectories for comparative evaluation rather than independently measured hardware truth. Accordingly, the semi-physical results are used primarily to assess relative performance, timing behavior, and robustness under realistic sensing and processing constraints.

**Figure 14:**
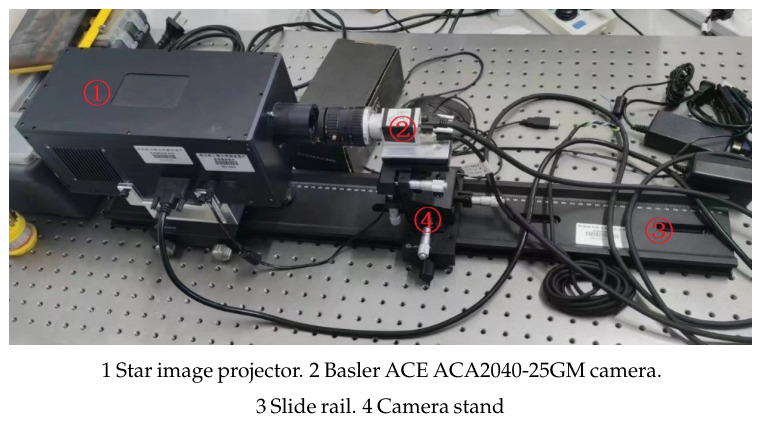
Semi-physical experimental setup. The reference trajectory denotes the commanded attitude profile.

Table 2 summarizes the device specifications, and Figure 14 illustrates the experimental setup. A simulated star field is played back through a projector and captured by a calibrated camera for star streak extraction and subsequent estimation. The reported runtime includes both front-end processing and back-end estimation. All algorithms are implemented in C++ and executed on a workstation equipped with an Intel^®^ Core™i5-8300H processor (Intel, Santa Clara, CA, USA).

For comparison, the proposed method is evaluated against SPKF, the proposed framework without the attitude-delay factor, and a “Proposed Lower Layer + MSCKF” configuration. MSCKF remains a widely adopted tightly coupled filtering framework in visual–inertial navigation [40,41]. Since it cannot directly process motion-blurred star streak observations, the optimized outputs of the proposed lower layer are used as common inputs, while only the upper-layer estimator is replaced by MSCKF.

Table 3 summarizes the quantitative results of the four estimation configurations. The proposed method achieves the best global attitude accuracy among all compared methods. The “Proposed Lower Layer + MSCKF” configuration uses the same lower-layer outputs while replacing only the upper-layer estimator, highlighting the benefit of the proposed factor graph optimization for global estimation. The local angular velocity results remain identical for the proposed method, the “Proposed Lower Layer + MSCKF” configuration, and the proposed method without the attitude-delay factor, since they share the same lower-layer optimization. In contrast, removing the attitude-delay factor noticeably degrades the global attitude estimation accuracy, whereas only marginal changes are observed in gyroscope bias estimation. This behavior is expected because the delayed attitude factor acts directly on the attitude states in the upper-layer optimization, thereby improving global attitude estimation under delayed observations. In contrast, the gyroscope bias is optimized through other constraints and is only indirectly influenced by the refined attitude estimates, resulting in marginal differences in bias errors.

**Figure 15:**
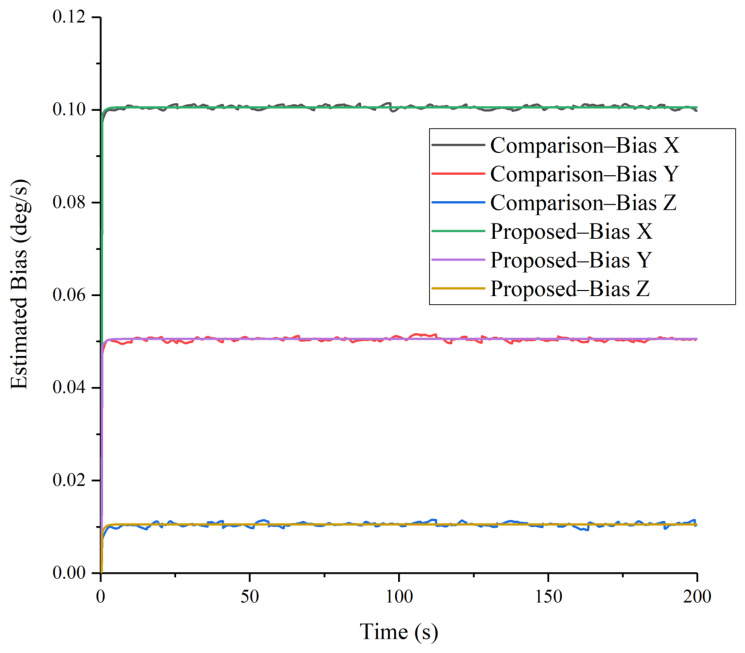
Gyroscope bias estimation errors for the proposed method and the SPKF.

Figure 15 further compares the gyroscope bias estimation performance. The proposed method exhibits faster convergence and lower steady-state bias error than SPKF, which is consistent with the quantitative results in Table 3. This indicates that the upper-layer optimization improves not only attitude consistency but also gyroscope bias estimation accuracy within the considered experimental duration.

**Figure 16:**
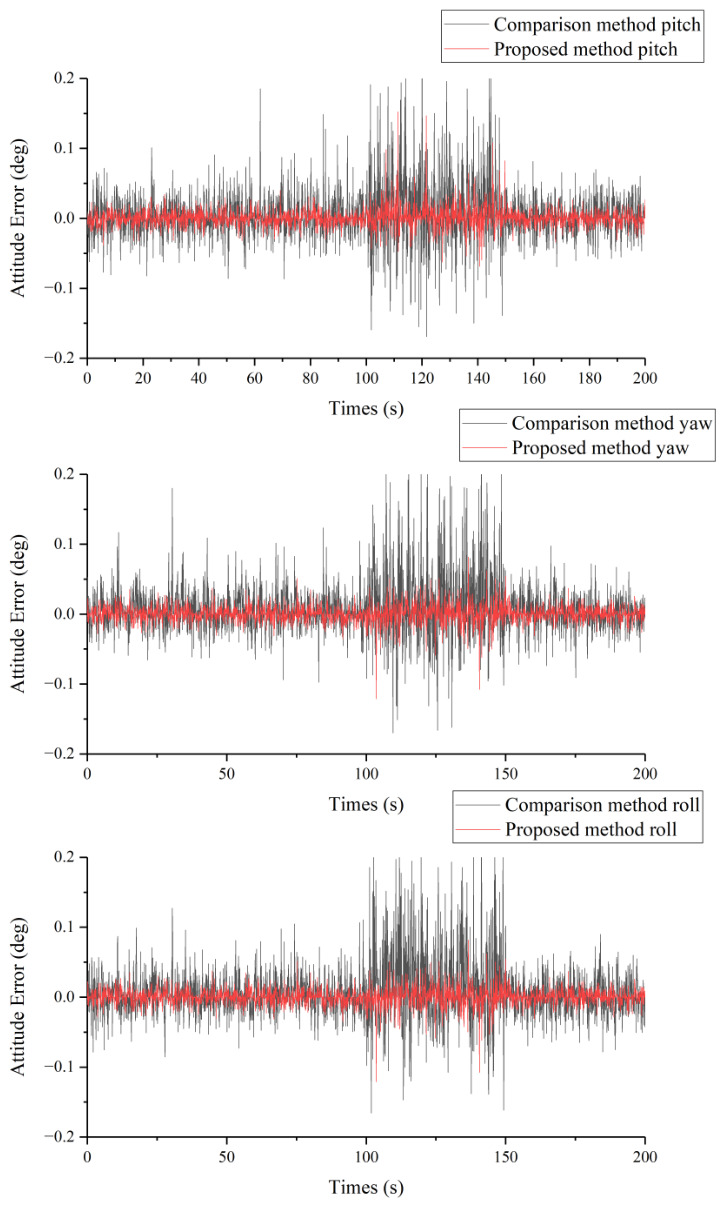
Attitude estimation error with temporary deactivation of the attitude factor (100–150 s).

Figure 16 compares the two methods during a 200 s experiment in which the attitude factors are intentionally disabled from 100 to 150 s. During the nominal interval (0–100 s), the proposed method already achieves substantially lower attitude error than SPKF, with a mean three-axis MAE/RMSE of approximately 0.0069/0.0101deg, compared with 0.0181/0.0251deg for SPKF.

When the attitude factors are removed, the errors of both methods increase; however, the proposed framework degrades more moderately. During the factor-disabled interval (100–150 s), the proposed method maintains a mean three-axis MAE/RMSE of approximately 0.0129/0.0192deg, whereas SPKF increases to 0.0489/0.0677deg, indicating significantly stronger error accumulation.

After the attitude factors are restored, the proposed framework rapidly returns to its nominal accuracy, while SPKF exhibits larger residual oscillations and slower recovery.

These results demonstrate that the proposed dual-layer framework maintains stable estimation performance under temporary loss of absolute attitude observations, while providing improved robustness and recovery capability under realistic sensing and processing constraints.

## 6. Conclusions

This paper presented a dual-layer factor graph framework for asynchronous star-tracker/IMU fusion in highly dynamic spacecraft attitude estimation. The proposed framework addresses the coupled challenges of motion blur, delayed star-tracker observations, asynchronous sensing, and inertial drift by combining a lower-layer local fusion graph with an upper-layer delay-aware global optimization graph, without relying on explicit interpolation, retrospective smoothing, or continuous-time temporal-state augmentation.

At the lower layer, IMU measurements are directly fused with single-frame blurred star streak observations to estimate exposure-related angular motion and construct timestamp-consistent representative observations. At the upper layer, delayed attitude constraints, propagated star-vector information, and inertial rotational constraints are jointly incorporated to refine the attitude trajectory with improved temporal consistency.

Simulation and semi-physical experimental results demonstrate that the proposed framework achieves improved attitude and angular velocity estimation accuracy, stronger robustness to delayed, sparse or intermittent star-tracker observations, and stable estimation performance under varying blur levels, timing misalignment, and high-dynamic rotational motion. In addition, the proposed method maintains practical computational efficiency for near-real-time onboard implementation.

Future work will focus on extending the framework toward more comprehensive multi-sensor fusion, improving the treatment of long-horizon temporal correlation, and further reducing computational complexity for resource-constrained onboard embedded platforms.

## Figures and Tables

**Table 1:** Nominal simulation parameters used in both local and global evaluations.

Parameter	Value
Angular velocity (default set)	[2,2,1]deg/s
Angular velocity (set 2)	[4,4,5]deg/s
Field of view (FOV)	10deg×10deg
IMU frequency	100 Hz
Resolution	500×500 pixels
Image update interval	0.1 s
Focal length	125 mm
Initial IMU bias	0.1deg/s
Epoch	J2000
Temporal optimization horizon	10 states
IMU noise mean / variance	$0.1\;\deg/s,\;0.04\;{\left(\deg/s\right)}^{2}$

**Table 2:** Sensor parameters.

Parameter	Value
Focal length	125 mm
Image resolution	1024×1024 pixels
Field of view (FOV)	12deg×12deg
Camera output rate	10 Hz
Initial gyro white noise std	[0.1,0.05,0.01]deg/s
Gyro white-noise variance	$\left[4\times 10^{-4},\;5\times 10^{-5},\;1\times 10^{-6}\right]\;{\left(\deg/s\right)}^{2}$
IMU output rate	100 Hz
Temporal optimization horizon	10 states
Experiment duration	200 s (2000 frames)

**Table 3:** Performance comparison of the proposed method and the SPKF.

Parameter	Proposed	SPKF	Proposed Lower Layer + MSCKF	Proposed (No Delay Factor)
Local Optimization (Angular Velocity)				
MAE (ω) [deg/s]	0.00481	0.00727	0.00481	0.00481
RMSE (ω) [deg/s]	0.00516	0.0123	0.00516	0.00516
Time per frame [s]	0.0585	N/A	0.0585	0.0585
Global Optimization (Attitude)				
MAE (attitude) [deg]	0.00692	0.01941	0.00886	0.0131
RMSE (attitude) [deg]	0.00997	0.02357	0.01247	0.0194
Processing time per dataset [s]	0.4751	0.2692	N/A	0.4598
Bias Estimation				
MAE (gyro bias) [deg/s]	0.00184	0.00421	N/A	0.00185
RMSE (gyro bias) [deg/s]	0.00261	0.00563	N/A	0.00269

## Data Availability

The data presented in this study are available from the corresponding author upon reasonable request.
